# Effective microorganisms, turmeric (*Curcuma longa*), and their combination on performance and economic benefits in broilers

**DOI:** 10.1016/j.heliyon.2022.e09568

**Published:** 2022-06-01

**Authors:** Chala Kinati, Nagasi Ameha, Meseret Girma, Ajebu Nurfeta

**Affiliations:** a Department of Animal Sciences, Ambo University, P O Box 19, Ambo, Ethiopia; b School of Animal and Range Sciences, Haramaya University, P O Box 138, Dire-Dawa, Ethiopia; c School of Animal and Range Sciences, Hawassa University, P O Box 5, Hawassa, Ethiopia

**Keywords:** Broiler, Economic efficiency, Effective microorganisms, Growth, Turmeric powder

## Abstract

The effects of effective microorganisms (EM), turmeric powder (TP), and their combination (EM-TP) on broiler performance, carcass characteristics, and economic benefit were studied in broilers fed a concentrate-based diet. A total of 192 chicks were assigned to four dietary treatments having CTL = control, EM = CTL+1 ml/lit effective microorganisms, TP = CTL+1%TP, EM-TP = CTL+0.5 ​ml/litEM+0.5%TP following a completely randomized design of 3 replications for each treatment. Concentrate was fed ad-libitum to all treatment groups. The feeding experiment lasted 42 days, 21 days for the starter and finisher phases each. The highest (P < 0.001) feed intake was observed when EM was fed as the sole additive and EM-TP during the starter period while the lowest (P > 0.05) value was for TP alone. There was no significant difference in feed intake during the finisher and the entire experimental period. The average daily gain for EM was higher (P < 0.05) than that of CTL and TP during the starter phase. However, during the finisher phase the average daily gain for EM-TP was greater (P < 0.05) than for TP and CTL. The greatest (P < 0.05) average daily gain was for EM-TP and EM during the entire period. The feed conversion ratio, performance index, mortality, and carcass characteristics were similar (P > 0.05) among treatments. The highest (P < 0.05) abdominal fat was observed in the control group. The finding indicates that a greater net return was earned from EM-TP while a lower net return was observed for TP. In conclusion, supplementation of EM (1 ​ml/lit) and the combination (EM-TP) at 0.5% each are better in terms of average body weight gain, the net return, and in decreasing abdominal fat.

## 1 Introduction

Since broilers are monogastric animals, which are typically raised in intensive production methods, are susceptible to several illnesses, which reduce their productivity (Kiczorowska et al., 2017). Growth promotants, antibiotics, and anti-coccidial medicines are frequently used to improve performance and reduce losses caused by disease-causing bacteria or pathogenic feed additives (Castanon, 2007; Hume, 2011). Excessive use of any antibiotic over time might cause bacterial populations to become resistant to the antibiotic, which can have a long-term effect. There is controversy about the use of growth promoters in animals destined for meat production. Broilers can be fed natural growth promoters like prebiotics, probiotics, synbiotics, enzymes, plant extracts, and other natural growth promoters without harming their performance (Borazjanizadeh et al., 2011). Prebiotics, probiotics, synbiotics, enzymes, plant extracts, and other natural growth promoters improve animal health by enhancing host mucosa immunity and increasing resistance to harmful bacterial colonization (Cheng et al., 2014).

Probiotics are monocultures or mixed cultures of living microorganisms that, when consumed, have a favorable impact on animal health by altering the gut microbiota quantitatively and qualitatively, as well as altering the immune system (FAO/WHO, 2001; Reid, 2016). Probiotics improve host animal performance by maintaining normal intestinal microflora through competitive exclusion and antagonism (Kizerwetter-Swida and Binek, 2009) and enhancing non-pathogenic facultative anaerobic and gram-positive bacteria that produce lactic acid and hydrogen peroxide (Higgins et al., 2007). Moreover, this probiotic is suppressing intestinal pathogens and enhances the digestion and utilization of nutrients (Oelschlaeger, 2010). Such supplements are thought to alter the gut flora and promote intestinal absorption, resulting in improved performance (Sohail et al., 2012). In a broiler study, Hossain et al. (2012) observed that *A. canaliculatum* probiotics have high acid, bile, and heat resistance, and inhibit *E. coli* proliferation.

Turmeric rhizome (*Curcuma longa*/*Zingiberaceae*), more generally known as turmeric, is a widely used spice, food preservative, and coloring agent with biological and medical purposes (Chattopadhyay et al., 2004; Akbarian et al., 2012). *Curcumin*, *demethoxycurcumin*, *bisdemethoxycurcumin*, and *tetrahydro curcuminoids* are active compounds discovered in Turmeric (*Curcuma longa*) (Wuthi-Udomler et al., 2000). Curcumin is the essential bioactive component responsible for *Curcuma longa's* biological effect (Nouzarian et al., 2011).

The combination of probiotics and phytobiotics has been studied as an alternative to antibiotics in broiler feed (Ren et al., 2019). In vitro research with a combination of probiotics and phytobiotics showed that phytobiotics can support the growth of probiotic bacteria (Prakasita et al., 2019). It has been reported that probiotics and phytobiotics are more efficient when used together rather than using them separately (Yuanita et al., 2019). Likewise, EM and Turmeric feed additives as sole or in combination might have stronger effects on general health status, growth performance, and carcass characteristics (Chala et al., 2021). The goal of the study was to see how efficient microbes and turmeric powder, alone and in combination, affected broiler chicken growth, carcass features, and economic efficiency.

## 2 Materials and methods

### 2.1 Ethical approval

The protocols for this experiment, use, and care of broilers were carried out in accordance with the guidelines of the Animal Care and Use Committee of Haramaya University, Ethiopia.

### 2.2 Experimental site

The experiment was conducted at Haramaya University's poultry farm, which is located 515 km east of Ethiopia's capital, Addis Ababa. The elevation is 2006 m above sea level, with a latitude of 9^o^ 41′ north and a longitude of 42^o^ 4′ east (Mengesha et al., 2015). The area receives 790 mm of annual rainfall and has an annual mean temperature of 17 degrees Celsius, with mean minimum and maximum temperatures of 14 and 23.4 degrees Celsius, respectively (Kbrom et al., 2016).

### 2.3 Experimental bird management

The experiment was carried out for 42 days. Before the experimental birds were assigned, the experimental pen was cleaned and disinfected, and the floor was covered with a litter of teff straw (7 ​cm deep) and disinfected completely with hydrogen peroxide. As a source of heat and light, each pen was fitted with a 250-watt infrared bulb.

*A day* before the birds were to be placed, a circular plastic feeder and waterer were placed in each of the pens. A total of 192 Cobb 500-day-old unsexed broiler chicks were acquired and transferred to Haramaya University's chicken farm from Alama farm in Bishoftu, Ethiopia. Water was provided ad libitum, and measured amount of the experimental ration were provided twice a day at 8:00 and 16:00 h on an ad-libitum basis (∼15 percent refusal). Every morning, the refusals were recorded to calculate feed intakes. Bodyweight was assessed using sensitive balance at the beginning, at weekly intervals during the experimental period, and at the end of the feeding study. Throughout the trial, the regular bio-security approach was followed. Newcastle and Gumboro (infectious bursal disease) vaccines were given to the chicks.

### 2.4 Dietary treatment and experimental design

Soybean meal, noug seed cake, corn grain, wheat short, turmeric, vitamin premix, di-calcium phosphate, limestone, salt, and lysine and methionine were added to broiler rations.

Corn grain, noug seed cake, and turmeric were hammer-milled to a 5 mm sieve size and mixed with. Lysine, methionine, di-calcium phosphate, and vitamin premix were added to the feed during mixing without hammer milling. Representative samples of soybean meal, noug seedcake, maize grain, wheat short, and turmeric were analyzed for dry matter, crude protein, ether extract, crude fiber, and ash following the method of AOAC (2000). Calcium and phosphorus content was analyzed by atomic absorption spectroscopy and spectrophotometer method, respectively (AOAC, 1998) (Table 1).

**Table 1:** Feed ingredient chemical composition and experimental diets (% dry matter, except DM and ME).

Feed ingredients	Chemical Composition							
	DM %	CP	EE	Ash	CF	Ca	P	ME (kcal/kg DM)
Maize	90.50	8.78	4.28	4.73	2.97	0.03	0.83	3736.30
Wheat short	91,00	15.00	3.84	5.02	9.87	0.19	0.78	2980.30
Soybean meal	93.80	39.69	8.53	6.37	6.04	0.34	0.66	3617.90
Noug seedcake	93.00	30.80	7.84	9.38	18.50	0.33	0.32	2314.30
Turmeric powder	89.37	8.63	3.99	4.15	1.65	0.28	0.15	3852.40

DM = Dry mater CP = crude protein; EE = ether extract; CF = crude fiber; ME = Metabolizable energy; Ca = Calcium; P = phosphorus.

Weljijie PLC in Bishoftu, Ethiopia, supplied enough active EM1 in a plastic jar, which was transported to Haramaya University's poultry farm and stored properly. The EM preparations used in this study were made in accordance with EMROSA (2003) criteria. This EM has substantial populations of lactic acid bacteria (*Lactobacillus and Pedicoccus*) in suspensions of 1 × 10^5^ CFU/ml, yeast (*Saccharomyces*) in suspensions of 2 × 10^6^ CFU/ml, and fewer numbers of photosynthetic bacteria, actinomyces, and other species (Matthew, 2002). The proposed activated EM1 (1 ml/L) was added immediately to chlorine-free pure drinking water.

The isocaloric and isonitrogenous treatment rations were created using Feed Win software to suit the nutrient requirements of broilers (NRC, 1994). As a result, starter treatment rations comprised around 3000 kcal ME/kg DM and 22% crude protein, while finisher treatment rations contained 3100 kcal ME/kg DM and 19% crude protein. The starter phase lasted until three weeks of age, and the finisher phase lasted from four to six weeks. Separate diets were formulated for the starter and finishers phase (Table 2).

**Table 2:** The proportion (%) of ingredients and their respective chemical composition of experimental diets.

Ingredients	Starter (1–3 weeks)				Finisher (4–6 weeks)			
	CTL	EM	TP	EM-TP	CTL	EM	TP	EM-TP
Maize	59	59	59	59	61.7	61.7	61.7	61.7
Wheat short	3	3	3	3	8	8	8	8
DL-methionine	0.5	0.5	0.5	0.5	0.1	0.1	0.1	0.1
Soybean meal	18	18	18	18	14	14	14	14
Noug seed cake	17	17	17	17	14	14	14	14
Vitamin premix	0.2	0.2	0.2	0.2	0.2	0.2	0.2	0.2
Salt	0.3	0.3	0.3	0.3	0.5	0.5	0.5	0.5
Limestone	0.5	0.5	0.5	0.5	0.5	0.5	0.5	0.5
L-Lysine	1	1	1	1	0.5	0.5	0.5	0.5
Dicalcium phosphate	0.5	0.5	0.5	0.5	0.5	0.5	0.5	0.5
Total	100	100	100	100	100	100	100	100
Turmeric (g/kg)	0	0	1	0.5	0	0	1	0.5
EM (ml/L)	0	1	0	0.5	0	1	0	0.5
**Composition** **(% DM basis)**								
DM	90.8	90.8	91.2	91.00	92.1	92.1	92.5	2.3
CP	21.08	21.08	21.43	21.65	18.99	18.99	19.31	19.51
ME (kcal/kg DM)	2968	2968	3005	2992	3095	3095	3133	3119
EE	4.10	4.1	4.51	4.35	4.31	4.31	4.43	4.28
CF	3.30	3.30	3.12	3.05	3.72	3.72	3.65	3.57
Ash	11.13	11.13	12.5	12.13	12.35	12.35	13.04	12.59
Ca	1.23	1.23	1.79	1.02	1.23	1.23	1.04	1.05
P	0.2	0.42	0.65	0.67	0.53	0.53	0.75	0.68

DM = dry matter; CTL = Control EM = Effective Microorganisms; TP = Turmeric Powder; CP = crude protein; ME = Metabolizable energy; Ca = Calcium; P = phosphorus.

The chickens were assigned to four dietary treatments having CTL = control/no additive, EM = CTL +1 ml/lit effective microorganisms, TP = CTL +1% TP, EM-TP = CTL + combination of 0.5 ml/lit EM + 0.5% TP following a completely randomized design of 3 replications for each treatment. Concentrate was provided ad -libitum to all treatment groups. Treatment groups consisted of 48 birds and randomly distributed to replicate groups (16 birds/replicate).

### 2.5 Data collection and measurements of parameters

#### 2.5.1 Feed intake, growth performance, and feed conversion ratio

The difference between the feed offered and the feed refused was used to calculate daily feed consumption. Every morning, the amount of food supplied, and the number of refusals were weighed and recorded. The average daily feed intake per bird was calculated as follows:

(1) $$\text{Mean}\;\text{daily}\;\text{feed}\;\text{intake}=\frac{\text{Mean}\;\text{total}\;\text{feed}\;\text{intake}}{\text{No}.\;\text{of}\;\text{experimental}\;\text{days}}$$

The bird's initial live weights were determined using sensitive balance at the start of the experiment. Following that, weekly average live weights were determined by weighing all of the birds in each pen before feeding and watering. The following formula was used to compute the average weekly weight increase (AWG) per bird using these live weights:

(2) AWG = W (T) - W (t_0_)

where: W (T) final body weight/bird (g), and W (t_0_) = initial body weight/bird (g).

The mean feed conversion ratio was determined by dividing the average daily feed intake (DFI) by a mean daily body weight gain (Lawrence and Fowler, 1998).

(3) FCR = DFI / Average daily body weight gain

#### 2.5.2 Performance index

(4) Performance Index (PI) = (BW gain per kg) / FCR) x 100 (North, 1981).

#### 2.5.3 Chick mortality

Over the growing and rearing seasons, mortality was observed in several regimens, and the percentage of mortality was computed using the following equation:

(5) $$Mortality\left(\%\right)=\frac{\text{Number}\;\text{of}\;\text{dead}\;\text{chicks}}{\text{Number}\;\text{of}\;\text{total}\;\text{chicks}}\times 100$$

#### 2.5.4 Slaughter procedure and carcass traits

At the end of the feeding trial, 2 birds were randomly selected from each replication for carcass characteristics. Broiler birds were starved for 13 h to ensure the complete emptying of the crop (Ari et al., 2013) and weighed immediately before slaughter (pre-slaughter weight). The birds' feathers were removed by a de-feathering machine after slaughter, and the carcass cuts and non-edible offal components were determined according to Kekeocha's (1985) protocol. After removing the blood and feathers, the dressed carcass weight was measured, and the dressing percentage was calculated by multiplying the proportion of dressed carcass weight to slaughter weight by 100. After removing the blood, feathers, lower leg, head, kidney, lungs, pancreas, crop, proventriculus, small intestine, caeca, large intestine, cloaca, and urogenital tracts, the weight of the eviscerated carcass was calculated. The eviscerated percentage was determined as the proportion of the eviscerated weight to slaughter weight multiplied by 100.

From eviscerated carcass weight, drumstick-thigh, breast, wings, back, and neck meat were separated and weighed, then their weight was divided by slaughter weight and multiplied by a hundred to determine percentage weights. Weighing the fat clipped from the proventriculus to the cloaca was used to determine abdominal fat. The heart, gizzard, and liver were among the edible offal (giblets) that were weighed in relation to the slaughter weight.

The following was calculated according to FAO (2001) procedures.

(6) Dressed weight = Drumstick-thighs + Wings + Breast + Ribs + Back + Heart + Liver + Gizzard + Neck + Feet + Head + Viscera (lung + pancreas + intestine)

(7) Eviscerated weight = Dressed weight – (Feet + Head + Viscera)

(8) $$\text{Dressed}\;\text{weight}\left(\%\right)=\frac{\text{Dressed}\;\text{weight}}{\mathrm{Pr}\text{e}-\text{slaughter}\;\text{weight}}\times 100$$

(9) $$\text{Eviscerated}\;\text{weight}\left(\text{\%}\right)=\frac{\text{Eviscerated}\;\text{weight}}{\text{Pre}-\text{slaughter}\;\text{weight}}\times 100$$

A sensitive balance was used to weigh portions of the gastrointestinal system (GIT) such as the crop, liver, gizzard, proventriculus, small intestine, caeca, and large intestine. The relative weight was computed by dividing the weight of GIT components by the total weight of the slaughtered animals. A measuring tape was used to determine the length of the pieces.

#### 2.5.5 Chemical analysis of the feed

Dry matter (DM), crude fiber (CF), ash, ether extract (EE), calcium, and phosphorus were analyzed in fed offerings. The Kjeldahl technique was used to determine nitrogen (N). By multiplying N by 6.25, the crude protein (CP) was calculated (AOAC, 1995). Spectrophotometer and atomic absorption spectroscopy were used to determine calcium and phosphorus, respectively (AOAC, 1998). The ME content of the experimental meals was determined using an indirect technique from the EE, CF, and ash using Wiseman's (1987) equation:

(10) ME (Kcal/kg DM) = 3951 + 54.4 EE - 88.7 CF - 40.8ash

#### 2.5.6 Economic efficiency analyses

The economic benefit of effective microorganisms and turmeric addition in the broiler (Cobb 500) feed were estimated using a partial budget analysis. The analysis considers the cost of feed (which is a variable cost) consumed by the chicks, as well as the cost of procuring chicks were assumed to be similar and selling prices of the broiler's carcasses were based on average live weight (kg) for all the treatments respectively, while other costs (labor cost, vaccination cost, house rent, electricity cost) were assumed to be similar for all the treatments. The cost of feed consumed per bird was obtained by multiplying feed consumed per bird by feed cost per kilogram of feed.

The difference between the feed expense to formulate each treatment diet and the pricing of live birds was used to calculate variable costs. The amount of money left after total variable costs (TVC) were deducted from the total rate of return was computed as net income (NI) (TRR) (Knott et al., 2003).

(11) NI = TRR – TVC

The difference between the change in the total rate of returns (ΔTRR) and the change in total variable costs (ΔTVC) was used to compute the change in net income (ΔNI):

(12) ΔNI = ΔTR - ΔTVC

The marginal rate of return (MRR) measures the increase in ΔNI which was generated by each additional unit of the level of supplement or expenditure (ΔTVC) (Knott et al., 2003):

(13) MRR = ΔNI / ΔTVC

### 2.6 Statistical analysis

The data were subjected to an analysis of variance test for each parameter using general linear models' techniques of SAS statistical package version 9.3's (SAS, 2010). At P < 0.05, Duncan's multiple range test was employed to discover differences between the treatment means (Duncan, 1955).

The model used was: Y_*ij*_ = μ + α_*i*_ + ε_*ij.*_ Where: Y_ij_ = the j^th^ the observation with treatment i, μ = overall mean, α_i_ = the i^th^ treatment effect, ε_ij_ = the random error of variation normally and independently distributed.

## 3 Results

### 3.1 Chemical composition

The proximate analysis of the feed ingredient and additives used in the experimental diets are presented in Table 1. Turmeric has the highest calculated metabolizable energy (3852.4 kcal/kg DM) content than the other major feed ingredients while, CP (8.63%), ash (4.15%), and CF (1.65%) content were lower than the other major feed ingredient.

### 3.2 Growth performance

The total feed intake, body weight gain, average daily gain, feed conversion ratio, performance index, and mortality percentage of broilers during the starter and finisher periods are shown in Table 3. The highest (P < 0.001) feed intake was for EM and EM-TP diets during the starter period while the lowest values (P < 0.05) were observed for TP. There was no significant difference (P > 0.05) in feed intake during the finisher and the entire experimental period.

**Table 3:** Effects of dietary inclusion of effective microorganisms’ turmeric and its combination on growth performance of broilers.

Parameter (g/bird)	Treatment				SEM	P-value
	CTL	EM	TP	EM-TP		
**Feed Intake**						
Starter	1053.90^b^	1127.90^a^	978.00^c^	1148.70^a^	21.29	0.001
Finisher	2787.10	3396.30	2773.20	3035.40	178.84	0.119
Entire period	3841.00	4524.10	3751.20	4184.10	188.10	0.068
Initial body weight	43.30	43.73	43.61	43.80	0.43	0.840
**Final body weight**						
Starter	354.51	421.27	362.52	418.14	25.72	0.207
Finisher	1753.40^b^	1972.10^a^	1678.60^b^	2001.10^a^	63.52	0.016
**Bodyweight gain**						
Starter	311.21	377.54	318.91	374.34	25.70	0.210
Finisher	1398.9	1550.9	1316.0	1582.9	75.30	0.108
Entire period	1710.10^b^	1928.37^a^	1634.99^b^	1957.30^a^	60.71	0.004
**Average daily gain**						
Starter	15.56^bc^	18.23^a^	15.09^c^	17.73^ab^	0.76	0.044
Finisher	82.26^bc^	92.27^ab^	78.68^c^	93.89^a^	3.08	0.020
Entire period	48.91^b^	55.25^a^	46.89^b^	55.81^a^	1.44	0.004
**Feed conversion ratio**						
Starter	3.58	2.96	3.09	3.08	0.17	0.134
Finisher	1.61	1.76	1.70	1.54	0.12	0.617
Entire period	2.60	2.36	2.39	2.31	0.09	0.166
**Performance index**						
Starter	9.36	13.04	10.26	12.07	0.95	0.089
Finisher	108.40	112.34	99.86	128.14	10.73	0.360
Entire period	117.75	125.38	110.12	140.21	10.41	0.280
**Mortality %**						
Starter	8.33	4.17	4.17	4.17	3.76	0.819
Finisher	4.17	10.42	4.17	6.25	3.13	0.487
Entire period	12.50	14.58	8.33	10.42	4.77	0.812

^abc^ Within a row means with different letters are significantly different at P < 0.05.

CTL = Control, EM = Effective microorganisms, TP = Turmeric powder, EM-TP = Combination of effective microorganisms and turmeric powder.

The final body weights were similar (P > 0.05) among treatments during the starter phase. However, the highest (P < 0.05) final body weight was for EM and EM-TP during the finisher phase. The average daily gain for EM was higher (P < 0.05) than that of control and TP during the starter phase. However, during the finisher phase the ADG for EM-TP was higher (P < 0.05) than for TP and control. The highest ADG was for EM-TP and EM for the entire period. The FCR, performance index and mortality were similar (P > 0.05) among treatments.

### 3.3 Carcass characteristics

The carcass characteristics did not show a significant difference among the treatments (Table 4). The highest (P < 0.05) abdominal fat was detected in the control group.

**Table 4:** The effect of dietary inclusion of effective microorganisms, turmeric powder, and their combination on carcass component of broiler chicken.

Parameters	Treatments					
	CTL	EM	TP	EM-TP	SEM	P-value
Slaughtering Weight(g)	1779	1808	1747	1911	84.51	0.57
Dressed carcass wt. (g)	1699	1622	1609	1712	66.11	0.70
Dressing percentage	91.60	89.79	92.29	89.61	1.31	0.43
Eviscerated weight (g)	1015	1131	989.0	1023	72.99	0.56
Eviscerated percentage	57.11	63.07	56.64	53.64	4.52	0.55
Breast (g)	370.3	415.6	389.5	348.6	37.52	0.646
Breast %	526.6	628.4	586.0	508.1	73.79	0.657
Thighs (g)	184.3	189.1	154.0	183.6	13.34	0.300
Thighs %	261.4	285.4	231.9	263.4	24.86	0.536
Drumsticks (g)	151.6	168.0	142.1	156.3	11.75	0.508
Drumistics %	215.1	253.8	213.9	224.0	22.39	0.582
Wings (g)	6.520	74.52	60.77	70.50	3.94	0.172
Wings %	97.29	112.3	91.39	100.7	7.29	0.298
Back (g)	132.9	169.7	132.1	153.2	17.46	0.416
Back %	189.0	257.9	199.1	219.8	32.52	0.492
Neck (g)	70.95	62.50	53.17	61.02	4.9	0.164
Neck %	100.7	94.44	79.96	88.30	9.93	0.529
Gizzard (g)	57.51	61.99	61.23	55.40	3.19	0.459
Gizzard %	81.56	93.27	91.99	79.38	5.88	0.297
Liver (g)	38.72	39.62	40.01	44.06	2.63	0.525
Liver %	55.03	59.36	59.94	63.69	5.07	0.700
Heart (g)	11.19	12.10	9.32	11.04	0.80	0.179
Heart %	15.89	18.31	14.03	15.80	1.57	0.352
Abdominal fat (g)	41.20^a^	20.00^c^	28.12^b^	23.58^bc^	2.05	0.000
Abdominal fat %	58.49^a^	29.99^c^	42.24^b^	33.74^bc^	3.30	0.001

^abc^ Within a row means with different letters are significantly different at P < 0.05.

DP = Dressing percentage, EP = Eviscerated percentage, CTL = Control, EM = Effective microorganisms, TP = Turmeric powder, EM-TP = combination of effective microorganisms and turmeric powder, SEM = Standard error of the mean, P = Probability, Wt = Weight.

### 3.4 Economic efficiency

**Figure 1:**
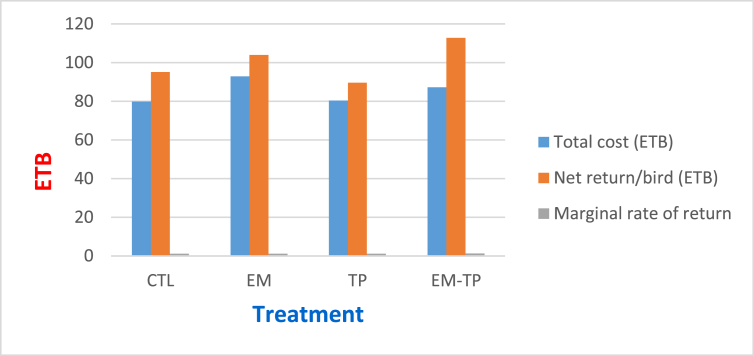
Economic efficiency of inclusion of EM and TP feed additives in broiler chickens.

According to the partial budget analysis, the profitability of broilers supplemented with EM and turmeric is given in Figure 1. The finding indicates that a higher net return (112.73 ETB) was earned from the combination of EM (0.5 ml/lit) and TP (0.5%) followed by EM alone as an additive (104.03 ETB). Lower net return (89.63 ETB) was observed for TP at 1% level followed by the control group (95.1 ETB).

## 4 Discussion

### 4.1 Chemical composition

The CP content of turmeric (8.63%) was lower but that of ME content was higher in turmeric than in the other feed ingredient used in the current experiment. This result is similar to the finding of Ikpeama et al. (2014) who reported that turmeric contains 8.92 percent moisture, 2.85 percent ash, 4.60 percent crude fiber, 6.85 percent fat, 9.40 percent crude protein, and 67.38 percent carbohydrate. Moreover, Youssef et al., (2014) reported turmeric as having 67.91 percent carbohydrates, low contents of fat (2.46 percent), 4.02 percent fiber, and 9.34 percent protein.

### 4.2 Effect of effective microorganisms and turmeric powder on growth performance

The greater growth rate in probiotic-treated broilers could be attributable to increased feed intake (Lei et al., 2015) and enhanced feed usage efficiency as compared to untreated broilers (Zhang and Kim, 2014). The current finding indicates that EM and TP-EM resulted in better feed intake during the starter phase, and they improve FBW during the finisher phase compared to the other treatment. In this regard, Bai et al. (2013) observed that probiotic feeding in poultry promotes feed intake during the starter (early) phase (Bai et al., 2013), while others affect body weight during the grower-finisher (later) phase (Abdel-Rahman et al., 2013; Chawla et al., 2013) compared to the control. Other research, on the other hand, indicated that probiotics improved broiler growth throughout the production cycle as compared to the control group (Mookiah et al., 2014). The dynamics of the gut microbiota may be the likely cause of this variability in probiotic efficacy at different stages (FAO, 2016). Choosing the proper EM strain for the right attribute (parameter) for this particular growth period will have to be identified. The other cause could be enhanced enzyme activity in the colon, as well as greater feed intake and increased digestion and absorption of nutrients. According to Jin et al. (2000), *Lactobacillus acidophilus* supplementation at a rate of 2 × 10^6^ cfu/g of maize-soybean-based diet boosted amylase activity in the small intestine of poultry by 42 percent.

In this study, BWG was increased by EM and EM-TP than in the other treatment group for the entire experimental period. The result agrees with Hossain et al. (2012) who indicated that the final body weight and body weight gain at the finisher and total periods were higher in the *Alisma canaliculatum* with probiotics at 0.5% compared to the negative control; basal diet and *Alisma canaliculatum* with probiotics at 1%. Broilers fed probiotics *Lactobacillus*, *Bifidobacterium*, coliforms, and Clostridium species gained significantly more body weight, according to Song et al. (2014). Similarly, Mondal et al. (2015) reported that the body weight gain in broilers fed a diet containing turmeric powder at the level of 0.5% was higher than birds received 1.5%, 1%, and 0% turmeric powder. The significant increase in body weight for 0.5% turmeric powder and 0.5 ml/lit additives may be due to the synergetic effect of optimum antioxidant activity of turmeric (*Curcuma longa*) that can stimulate protein synthesis by the bird's enzymatic system and effective microorganism in the intestine that stimulates the production of bird's enzymatic activity.

EM enhanced average daily body weight gain during the starter phase, but throughout the study, both EM and EM-TP improved BWG more than the other treatment, which is similar to the finding of Zhang et al. (2005) and Paryad and Mahmoudi (2008), who found that supplementing broiler rations with probiotic yeast (*Saccharomyces cerevisiae*) improved body weight gain and feed conversion ratio. *Saccharomyces cerevisiae* improved feed/gain ratio and body weight gain, Gudev et al., (2008), Patane et al. (2017). One of the critical functions of EM in metabolic function is to promote a healthy or pathogen-free gastrointestinal tract environment for endogenous enzymes to properly break down the nutrients of the experimental rations, as well as to reduce competition for energy and nutrients between probiotic and pathogenic microorganisms (Kassech et al., 2019). Wang et al. (2015) found that supplementing broilers with dietary turmeric rhizome extract increased ADG and FCR during the finishing phase. Curcumin's favorable benefits on broiler growth performance may be attributable to increased secretions of the enzyme's amylase, trypsin, chymotrypsin, and lipase (Platel and Srinivasan, 2000).

### 4.3 Effect of effective microorganisms and turmeric powder on carcass traits

Supplementation of EM, TP, and the combination (EM-TP) had no effect on carcass traits except the abdominal fat in the current experiment. There were no documented changes in the relative weights of internal organs from broilers fed *Lactobacillus* spp. and medicinal herbs (Hernandez et al. 2004; Awad et al., 2010). The current findings are congruent with those of Santoso et al. (2001), who found that specific bacteria in the gastrointestinal tract of birds inhibited cholesterol and bile acid absorption. It is possible that the microorganisms in the probiotic-yeast combination helped to reduce fat absorption and accumulation in the abdomen. Probiotics have been shown to lower fat content and improve carcass characteristics in broiler chickens (Fouad and El-Senousey, 2014; Shabani et al.,2012). In addition, Mondal et al. (2015) found that adding turmeric powder to the broiler feed reduced the fat content of the broiler when compared to the control group. Moreover, Yonatan et al. (2017) found that including turmeric powder in broiler meals at a rate of 1–2 g/kg reduced fat deposition in the abdomen area of broilers when compared to the control group. The quantity of belly fat was lowest in the 0.5 percent turmeric powder diet compared to the control diet (Mondal et al., 2015).

### 4.4 Economic efficiency of broiler chickens

Economic efficiency was defined as the net revenue per unit of feed cost computed from input-output analysis. In the current study, the higher net profit from the combination of EM and TP at 0.5% level and EM at 1 ​ml/lit was in contract with the result of Swain et al. (2012) who observed higher net profit and benefit: cost ratio in broilers fed a 1.0 g/kg diet containing probiotics and yeast. Another study showed that supplementation of probiotic and enzyme mixture in chick diet at 400 mg/kg increased the profit from 4.8 to 8.6% (Swain et al., 2009). In contrast, Kefali et al. (2007) reported that probiotic supplementation did not generate any additional revenue under the market conditions. Therefore, in this study economic data clearly indicated that EM at 1 ​ml/lit and the combination (EM-TP) at 0.5% for each additive are better for maximizing profitability.

## 5 Conclusion

Supplementation of EM (1 ​ml/lit) and the combination (EM-TP) at 0.5% each are better in terms of average body weight gain, net return, and decrease abdominal fat. Therefore, the result of the current study recommends that dietary inclusion of EM (1 ml/lit) and EM-TP (0.5 percent each) can be utilized as an additive in improving body weight gain and lowering abdominal fat in broilers, as well as improving profitability.

## Declarations

### Author contribution statement

Chala Kinati: Conceived and designed the experiments; Performed the experiments; Analyzed and interpreted the data; Wrote the paper.

Nagasi Ameha; Meseret Girma; Ajebu Nurfeta: Conceived and designed the experiments; Performed the experiments; Contributed reagents, materials, analysis tools or data.

### Funding statement

This work was supported by the 10.13039/501100003081Ethiopian Ministry of Education and the 10.13039/501100004535Ethiopian Institute of Agricultural Research.

### Data availability statement

Data will be made available on request.

### Declaration of interest’s statement

The authors declare no conflict of interest.

### Additional information

No additional information is available for this paper.
